# The Dynamic Process of Interspecific Interactions of Competitive Nitrogen Capture between Intercropped Wheat (*Triticum aestivum* L.) and Faba Bean (*Vicia faba* L.)

**DOI:** 10.1371/journal.pone.0115804

**Published:** 2014-12-26

**Authors:** Chunjie Li, Yan Dong, Haigang Li, Jianbo Shen, Fusuo Zhang

**Affiliations:** 1 Center for Resources, Environment and Food Security (CREFS), China Agricultural University, Beijing, 100193, China; 2 College of Resources and Environment, Yunnan Agricultural University, Kunming, 650201, China; Northwest A&F University, China

## Abstract

Wheat (*Triticum aestivum* L.)/faba bean (*Vicia faba* L.) intercropping shows significant overyielding and high nitrogen (N)-use efficiency, but the dynamics of plant interactions have rarely been estimated. The objective of the present study was to investigate the temporal dynamics of competitive N acquisition between intercropped wheat and faba bean with the logistic model. Wheat and faba bean were grown together or alone with limited N supply in pots. Data of shoot and root biomass and N content measured from 14 samplings were fitted to logistic models to determine instantaneous rates of growth and N uptake. The superiority of instantaneous biomass production and N uptake shifted from faba bean to wheat with their growth. Moreover, the shift of superiority on N uptake occurred 7–12 days earlier than that of biomass production. Interspecific competition stimulated intercropped wheat to have a much earlier and stronger superiority on instantaneous N uptake compared with isolated wheat. The modeling methodology characterized the temporal dynamics of biomass production and N uptake of intercropped wheat and faba bean in different planting systems, which helps to understand the underlying process of plant interaction for intercropping plants.

## Introduction

In China, intercropping has been practiced for over 2000 years, and has made a great contribution to food security of northwest China, e.g. close to 43% of the total gain yield of Ningxia province was from intercropping in 1995 [1]. Intercropping is widely accepted as a sustainable practice, due to its yield advantage, high utilization efficiency of light and water, and pest and disease suppression [2]–[5]. Faba bean (*Vicia faba* L.) significantly facilitated phosphorus (P) uptake of neighbouring cereals by mobilization of sparsely soluble soil P [2], [6]. Sorghum (*Sorghum bicolor* (L.) Moench)/pigeonpea (*Cajanus cajan* (L.) Millsp) and millet (*Pennisetum glaucum* (L.) R.Br.)/groundnut (*Arachis hypogaea* L.) intercropping systems showed temporal and spatial complementarity for light, water and nutrients [4]. Rice blast was 94% less severe when intercropping a disease-susceptible variety with a resistant one compared with monocropped plants [5]. Among numerous intercropping systems, cereal/legume system is one of the most popular in low-input agriculture nowadays [7]. There are more than 100 combinations of intercropping systems practiced in China, of which 70% involve legumes [8].

Previous studies of intercropping focusing on belowground interactions have shown significant intercropping advantages of nutrient uptake [2], [8]–[11]. Some strategies have been observed to be involved in the facilitation in intercropping: Soil nitrate depletion of cereals enhanced N_2_ fixation of neighbuoring legumes [9], [12] and up to 5% of N fixed was transferred from the legume to the cereal directly [8]; Rhizosphere pH modification, carboxylate exudation, acid phosphatase secretion of legumes mobilized sparsely soluble P in soil and increased P uptake of neighbouring cereals [11], [13]. Species interactions in intercropping systems have usually been studied on the basis of yield and nutrient acquisition during a limited time of sampling, thus showing the final outcome of crop interactions, instead of the actual underlying processes. Moreover, most of experiments are restricted at best to only a few destructive samplings widely spaced in time even one harvest in the experimental end. Consequently, differences of yield or nutrient acquisition between monocropped and intercropped plants at the single harvest time cannot be explained fully without knowledge of the earlier, but unmeasured processes that caused the competition [14]. In the context of the definition of ‘competition’ [15], it is necessary to know how well individual plants and their neighbours capture soil resources, how effectively those resources are converted into biomass, and how that biomass is then used to capture more resources. Instantaneous rates of biomass production and N uptake (amount of increases of growth and N uptake by plant per day) can reveal the simultaneous resource fluxes into each competitor which can indicate periods when competitive interactions are most intense and probably most decisive in affecting the competitor's success [15]. Therefore, measuring instantaneous rates of resource capture can enhance our understanding how plants interact with each other at different sampling points in the growing season. Cumulative biomass production and resource uptake are the integrals of many instantaneous rates. Estimating those instantaneous rates repeatedly would reveal the temporal progression of those interactions as they unfold, which provide a direct link between the process of competition and the physiological activities of competing individuals [15].

Due to high potential for overyielding, wheat (*Triticum aestivum* L.)/faba bean intercropping system is widely used by famers, especially in south China, in which two plant species have the same sowing and harvesting time. The objective of this study was to assess the temporal dynamics of competitive N acquisition between intercropped wheat and faba bean by using a logistic model, and then understand the temporal dynamics of intra- and interspecific competition between different plant species.

## Materials and Methods

### Experimental design

A pot experiment was carried out in a glasshouse at China Agricultural University, Beijing, China. Air temperatures ranged from18–28°C. Evaporative cooling and shade cloth were used to prevent excessively high temperatures on sunny days. The soil used in the experiments was loam, and collected from fallow land of Shangzhuang Experimental Station, Beijing, China. Our collection was permitted by the Center for Resources, Environment and Food Security (CREFS), China Agricultural University. Air-dried soil was sieved (2 mm). Each pot (210×170 mm) was filled with 2 kg soil. The soil had an organic carbon concentration of 10.1 g kg^−1^, a total N concentration of 0.6 g kg^−1^, a mineral N concentration (Nmin) of 18.3 mg kg^−1^: nitrate N of 15.1 mg kg^−1^ and ammonium N of 3.2 mg kg^−1^, Olsen-P of 6.7 mg kg^−1^, NH_4_Ac-exchangeable K of 74.1 mg kg^−1^, and a pH in CaCl_2_ of 7.8 (the ratio of soil to CaCl_2_ solution is 1∶2.5). Basal nutrients were added in a solution to soil (mg kg^−1^ soil): N, 100 as Ca(NO_3_)_2_, P, 150 as KH_2_PO_4_, K, 188.7 as KH_2_PO_4_. The added nutrients were thoroughly mixed with the soil by shaking. There were no other micronutrients added and no any deficiency symptoms observed on plants at each sampling.

**Figure 1:**
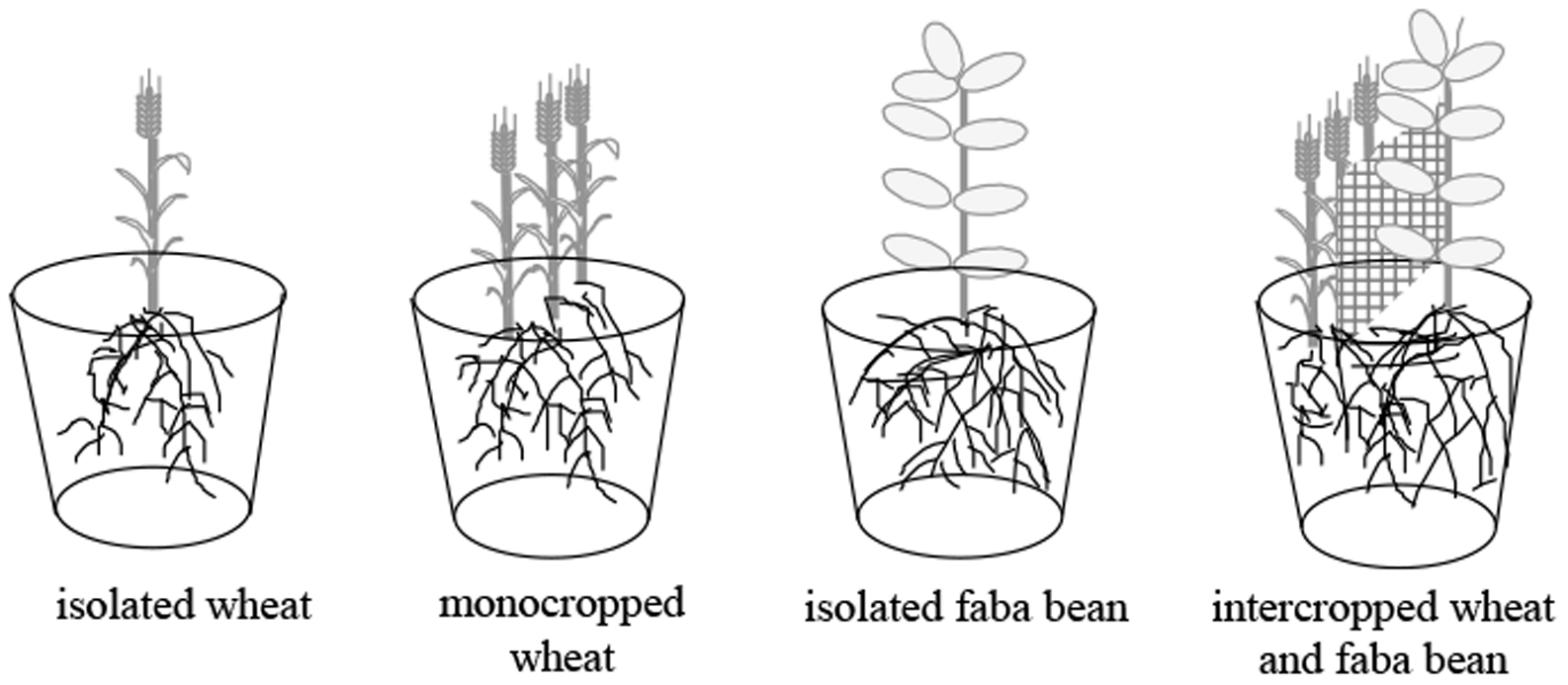
Schematic representation of different plant combinations.

Six germinated seeds of wheat (*Triticum aestivum* L cv. Yunmai-42) and two seeds of faba bean (*Vicia faba* L. cv. 89–147) were grown separately or together in one pot. Plants were thinned to three per pot for wheat and one for faba bean based on plant density in field at 11 days after sowing (DAS) (Fig. 1). Another treatment was that two germinated seeds of wheat were grown in one pot and then thinned to one plant at 11 DAS, which was free of competition and was aimed to set a comparison with other treatments that suffered with intra or interspecific competition. The pots were watered regularly to 70% of field capacity (21%, w/w). There were 14 destructive samplings, taken every 3–5 days from 15 to 62 DAS. Each treatment was replicated for 3 times. Window screens were used to prevent one plant from over-topping the other to avoid competition for light.

### Sampling and data collecting

There were 14 destructive samplings taken every 3–5 days from 15 to 62 DAS when the wheat has been grain-filling and faba bean has been pod-filling. At each sampling, shoots were removed by cutting at the soil surface and roots were removed from the soil by washing them carefully. When washing the roots, deionized water and sieve with 1 mm aperture were used. All sampled material was oven-dried at 76°C for 72 h before weighing. Nitrogen concentration of plant material was determined by wet digestion with a mixture of concentrated H_2_SO_4_ and H_2_O_2_ using the micro-Kjeldahl procedure [16]. Nitrogen content was calculated as the product of N concentration and biomass in shoots or roots [16].

### Data analysis

The biomass (*Y*; mg) of one herbaceous plant grown from seed for several weeks is typically a sigmoid function of time, *t* [17]. For each daily interval, *Δt*, *Y* was computed using a discrete two-parameter form of the logistic equation as following [15]. The logistic equation: 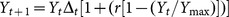
(*r*, the rate constant (d^−1^) for biomass production; *Y*_max_, a maximum to which *Y* tends asymptotically) [15] was used to separately fit the data of shoot and root biomass and N content by simultaneously adjusting *r* and *Y*_max_ to maximize *R^2^* with the SOLVER in Microsoft Excel 2010 [18]. The instantaneous rates were derived as the slopes of the logistic models fitted to the cumulative biomass or N content data, rather than directly from data.

## Results

### Cumulative biomass production and nitrogen uptake

**Figure 2:**
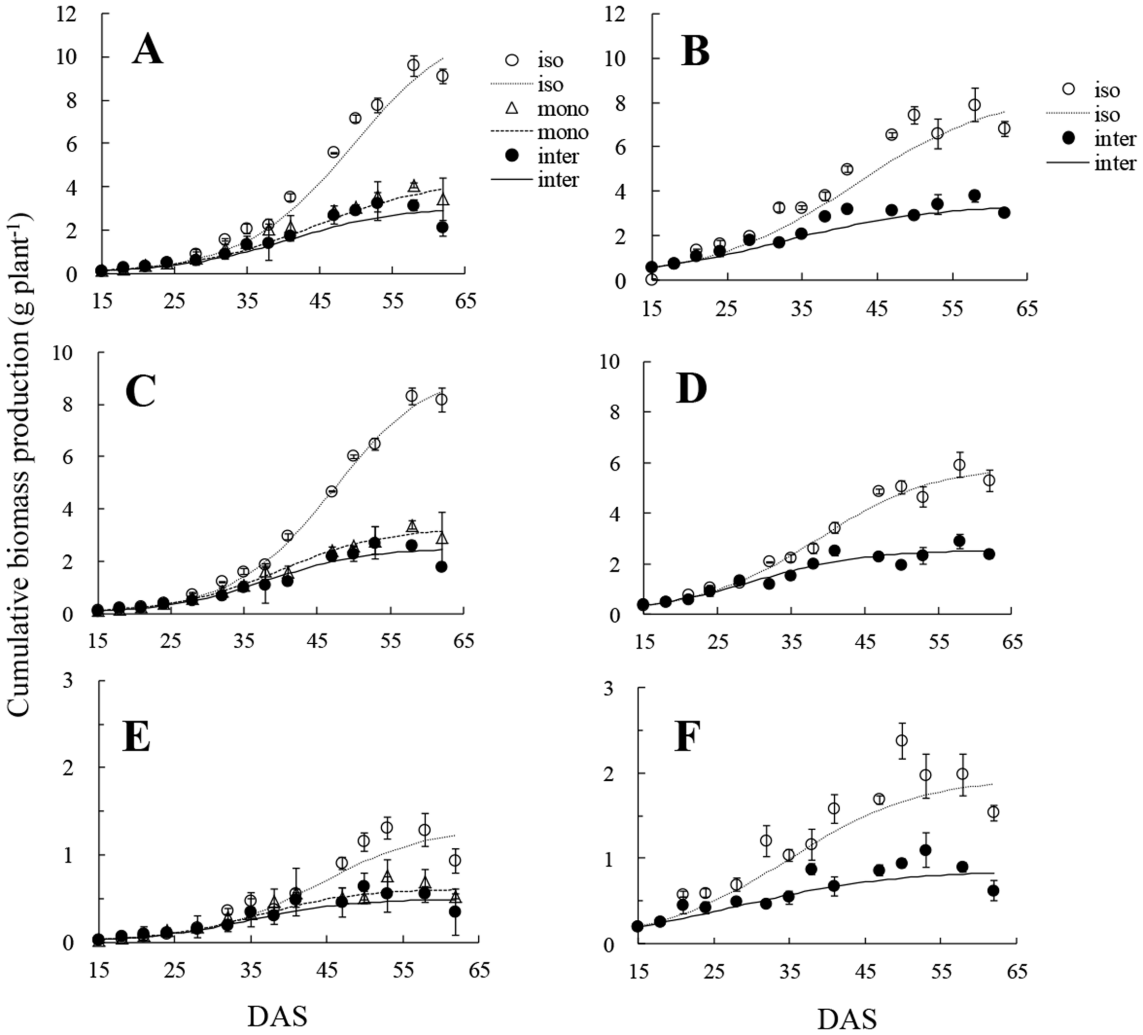
Cumulative biomass production. A, B: total plant biomass of wheat, and faba bean; C, D: shoot biomass of wheat, and faba bean; E, F: root biomass of wheat, and faba bean. iso: isolated plants; mono: monocropped plants; inter: intercropped plants. Open and solid circles, open triangles indicate the mean of actual data at each sampling. All values represent means±SE (n = 3). Curves are derived from the logistic equation using the mean of three replicates.

The maximum biomass of wheat or faba bean was influenced strongly by the presence of a neighbour and it differed between the two species. The maximum total plant biomass of intercropped wheat was 2.91 g plant^−1^, which was 71% less than that of isolated wheat, and 25% less than that of monocropped wheat (Table 1). Similarly, the maximum shoot biomass and root biomass of intercropped wheat was, respectively, 71% and 60% less than that of isolated wheat, and 23% and 18% less than that of monocropped wheat. The difference in shoot biomass among wheat plants grown in three cropping systems was larger than that of root biomass. The total plant, shoot and root biomass of intercropped faba bean was less than half of that of the isolated plants. Because a logistic model was fitted separately to total plant, shoot and root biomass, there were some differences between the sum of shoot and root biomass and total plant resulting from randomized errors. Divergences of biomass-production curves between isolated and monocropped or intercropped plants were detectable in total plant, shoot and root of wheat at *c.* 32 DAS after when the difference of measured biomass between isolated and monocropped or intercropped plants in total, shoot and root was significant (see S1 Table) (Fig. 2),. In contrast, faba bean had a much earlier divergence in root biomass at *c.* 18 DAS than in shoot biomass, at *c.* 28 DAS and in total plant biomass, at *c.* 28 DAS, and the difference of measured biomass between isolated and intercropped faba bean was significant from *c.* 32 DAS onwards (see S1 Table).

**Table 1:** The maximum biomass and nitrogen (N) uptake of wheat and faba bean estimated by a logistic model (Eqn 1).

		Wheat			Faba bean	
		Iso	Mono	Inter	Iso	Inter
Max. biomass (g plant^−1^)	Total	9.95	3.88	2.91	7.59	3.24
	Shoot	8.50	3.14	2.43	5.61	2.51
	Root	1.23	0.60	0.49	1.87	0.83
	Root/Shoot	0.14	0.19	0.49	0.33	0.33
Max. N uptake (mg plant^−1^)	Total	214.7	55.1	48.2	169.3	63.2
	Shoot	202.0	60.0	43.0	124.0	43.0
	Root	17.7	8.40	6.10	49.4	21.9

Note: A logistic model was fitted separately to the mean of three replicates of total plant, shoot and root. Iso indicates isolated plants; Mono indicates monocropped plants; Inter indicates intercropped plants.

**Figure 3:**
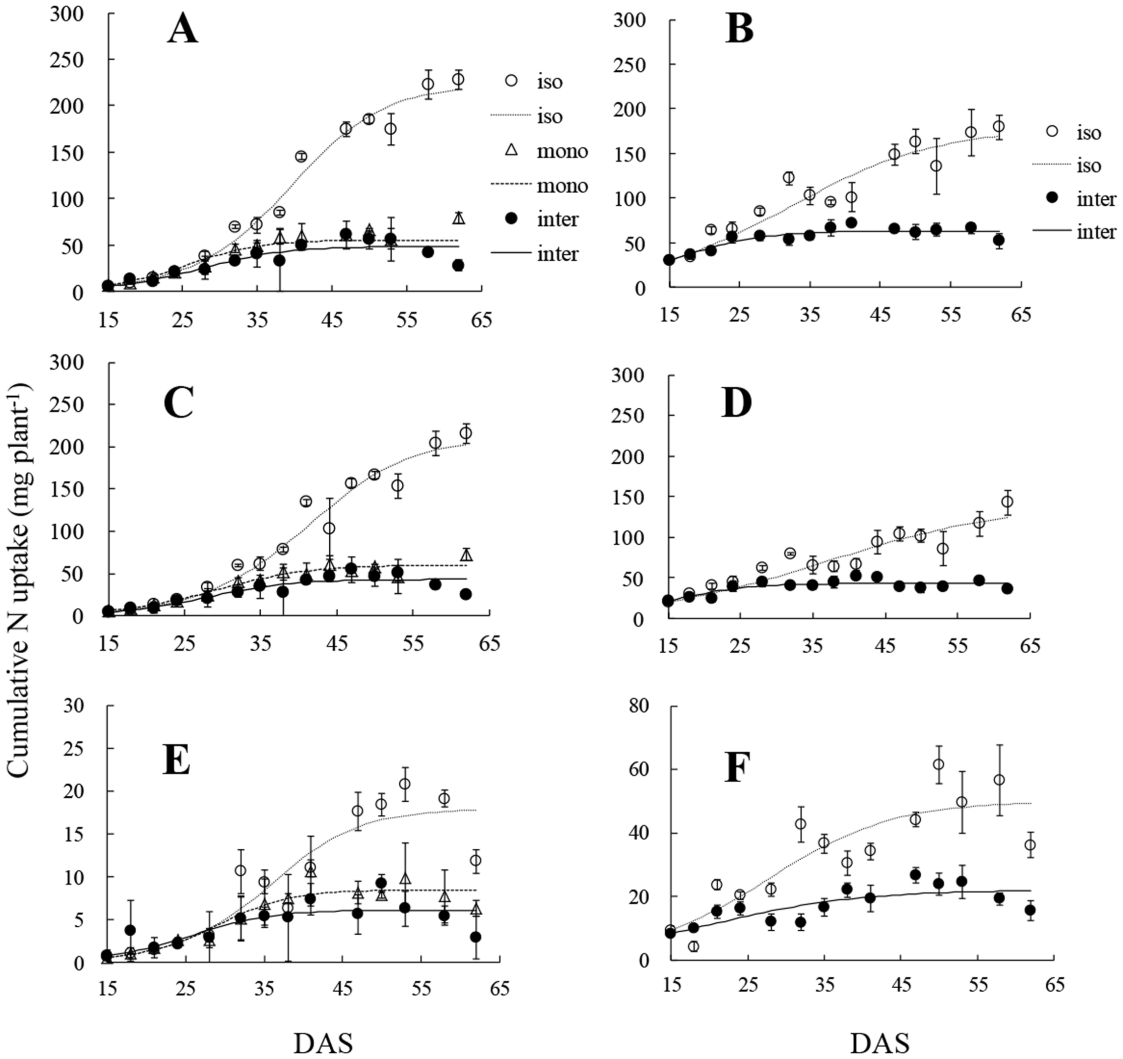
Cumulative nitrogen (N) uptake. A, B: total plant N uptake of wheat, and faba bean; C, D: shoot N content of wheat, and faba bean; E, F: root N content of wheat, and faba bean. iso: isolated plants; mono: monocropped plants; inter: intercropped plants. Open and solid circles, open triangles indicate the mean of actual data at each sampling. All values represent means±SE (n = 3). Curves are derived from the logistic equation using the mean of three replicates.

The maximum N uptake of both species was strongly influenced by interaction. Maximum N uptake by intercropped and monocropped wheat was only 22% and 26%, respectively, of that of isolated wheat (Table 1). In contrast to total plant and shoot N content in wheat, interaction of neighbouring faba bean had a weaker effect on maximum N content of root, which was 35% and 49% of that of isolated wheat in intercropped and monocropped wheat, respectively. Neighbouring wheat also reduced maximum N content of faba bean in total plant, shoot and root to 37%, 35% and 44% respectively. Wheat had a greater maximum N uptake per plant than faba bean in both isolated and monocropped systems. The cumulative curve of N capture by intercropped wheat diverged gradually from those of isolated plants, becoming obvious for total N uptake and shoot N content at *c.* 28 DAS, and for roots content at *c.* 31 DAS (Fig. 3). Also, the difference of N uptake between wheat in different treatments became significantly from *c.* 32, 35 and 41 DAS in total shoot and root (see S2 Table). Similarly, divergence of cumulative curves of N uptake between isolated faba bean and intercropped faba bean occurred at *c.* 28 DAS for total N uptake and shoot N content, and at *c.* 21 DAS for roots N content.

### Instantaneous biomass production and nitrogen uptake

**Figure 4:**
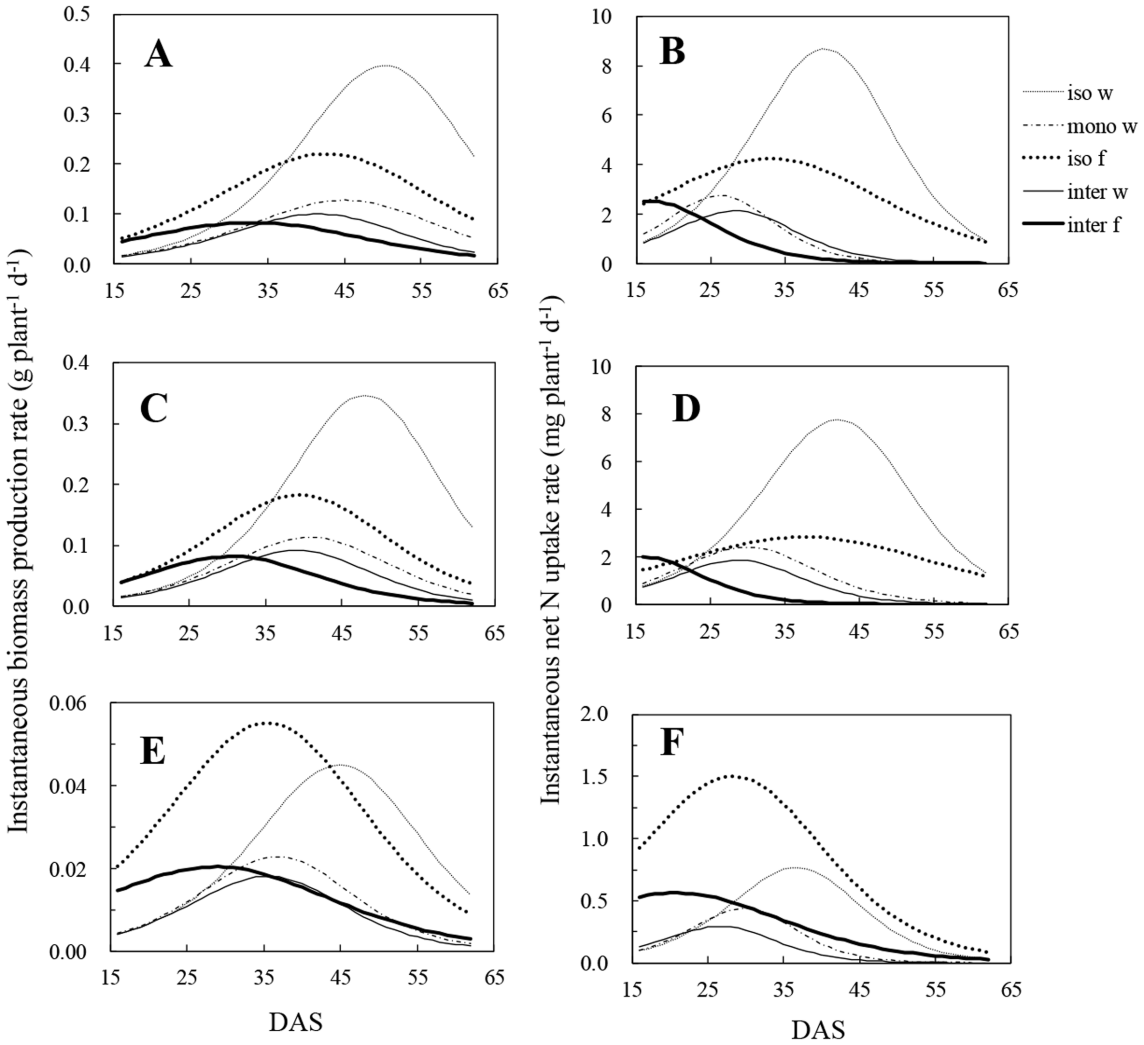
Instantaneous rate of biomass production (A, C, E), and instantaneous rate of nitrogen (N) uptake (B, D, F). A, B: total rate of biomass production and N uptake by wheat and faba bean of different planting systems. C, D: shoot growth rate and instantaneous per capita N uptake by shoot of wheat and faba bean. E, F: root growth rate and instantaneous per capita N uptake by root of wheat and faba bean. iso w: isolated wheat; mono w: monocropped wheat; iso f: isolated faba bean; inter w: intercropped wheat; inter f: intercropped faba bean.

Instantaneous biomass production rate in both wheat and faba bean showed a unimodal pattern (Fig. 4). Isolated wheat attained its maximum instantaneous biomass production rate (br_max_) for total plant biomass at 50 DAS which was delayed by 5 or 9 days compared with monocropped and intercropped wheat, respectively (Table 2). The time to attain br_max_ for shoot and root biomass of wheat was earlier than that for total plant biomass in all cropping designs. The br_max_ of intercropped wheat for total plant biomass was 0.10 g plant^−1^ d^−1^, which was only a quarter of that for isolated wheat. Intercropped faba bean reached a br_max_ of 0.08, 0.08 and 0.02 g plant^−1^ d^−1^ in total plant, shoot and root biomass, respectively, at 33, 30 and 29 DAS. Eight to ten days later, isolated faba bean also reached a br_max_ that was higher than that for intercropped faba bean, by 1.8, 2.3 and 1.5 times in total plant, shoot and root biomass, respectively. At harvest, the instantaneous biomass production rate of both intercropped wheat and faba bean was close to zero in total plant, shoot and root biomass.

**Table 2:** Times and rates of maximum instantaneous biomass production and nitrogen uptake by wheat and faba bean estimated by a logistic model (Eqn 1).

			Wheat			Faba bean	
			Iso	Mono	Inter	Iso	Inter
Biomass	Time (d)	Total	50	45	41	42	33
		Shoot	48	41	39	40	30
		Root	45	37	35	37	29
	Rate (g plant^−1^ d^−1^)	Total	0.40	0.13	0.10	0.22	0.08
		Shoot	0.35	0.11	0.09	0.18	0.08
		Root	0.05	0.02	0.02	0.05	0.02
N uptake	Time (d)	Total	40	26	28	33	15
		Shoot	42	30	28	37	15
		Root	37	30	27	28	21
	Rate (mg plant^−1^ d^−1^)	Total	8.66	2.74	2.12	4.22	2.52
		Shoot	7.75	2.40	1.86	2.82	1.99
		Root	0.77	0.44	0.29	1.50	0.56

Note: A logistic model was fitted separately to the mean of three replicates of total plant, shoot and root. Iso indicates isolated plants; Mono indicates monocropped plants; Inter indicates intercropped plants.

All plants attained the rate of maximum instantaneous N uptake (nr_max_) much earlier than the corresponding br_max_ in total plant, shoot and root biomass, regardless of cropping designs (Table 2, Fig. 4). Neighbouring plants reached the time of nr_max_ in total plant N content earlier than isolated plant by 12–14 days in wheat and 18 days in faba bean. However, the nr_max_ in total plant N uptake attained by wheat and faba bean was much reduced by the presence of neighbouring plants, to only 32% in monocropped wheat, 25% in intercropped wheat and 60% in intercropped faba bean of that of isolated plant, respectively. The nr_max_ of shoot and root N content was also less than that of isolated plants. At harvest, the nr_max_ of all plants in monocropping and intercropping designs was reduced to zero (Fig. 4).

### Cumulative biomass production and N uptake

**Figure 5:**
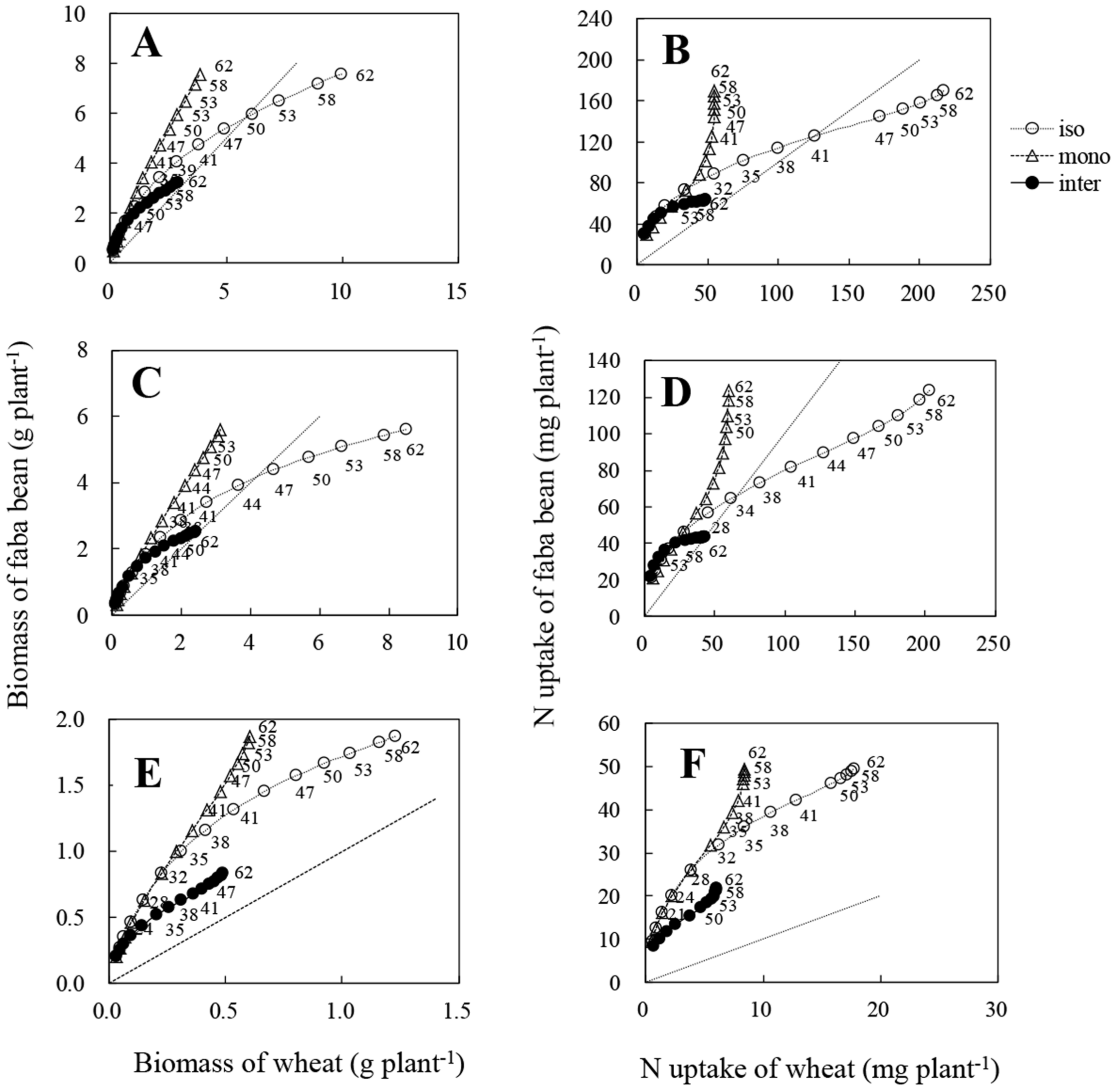
Trajectories of cumulative biomass production and cumulative nitrogen (N) uptake of total (A, B), shoot (C, D) and root (E, F) by isolated (iso: open circle and dashed curve), monocropped (mono: open triangles and dashed curve), intercropped (inter: solid symbols and continuous curve) wheat and according faba bean. Data was derived from the logistic equation using the mean of actual data at each sampling. Numbers near the curves represent the samplings time (day). The dashed line shows the 1∶1 relationship between faba bean and wheat.

Initially, the total cumulative biomass production of isolated faba bean was greater than that of isolated wheat until *c.* 50 DAS (Fig. 5A). This change-point was *c.*46 DAS for cumulative shoot biomass (Fig. 5C). In contrast, isolated faba bean always had a greater cumulative root biomass than that of isolated wheat (Fig. 5E). Intraspecific interaction strongly decreased biomass production of monocropped wheat, and resulted in no change-points for shifting growth superiority (the greater amount of biomass production or faster biomass production rate) during the growth period (Fig. 5A, C, E). In the intercropping system, competition between faba bean and wheat enhanced biomass loss of wheat. Intercropped faba bean also had a less than 50% biomass for total plant from *c.* 49 DAS, for shoot biomass from *c.* 51 DAS, and for root biomass from *c.*43 DAS, respectively.

Accordingly, the processes of cumulative total, shoot, root N content by faba bean and wheat were similar to that of biomass production in different cropping systems. However, the change-points for shifting growth superiority of cumulative total and shoot N content of isolated plants, respectively at *c.* 41 and 35 DAS, occurred 9–12 days earlier than that of cumulative biomass (Fig. 5).

### Dynamic trajectories of biomass production and N uptake

**Figure 6:**
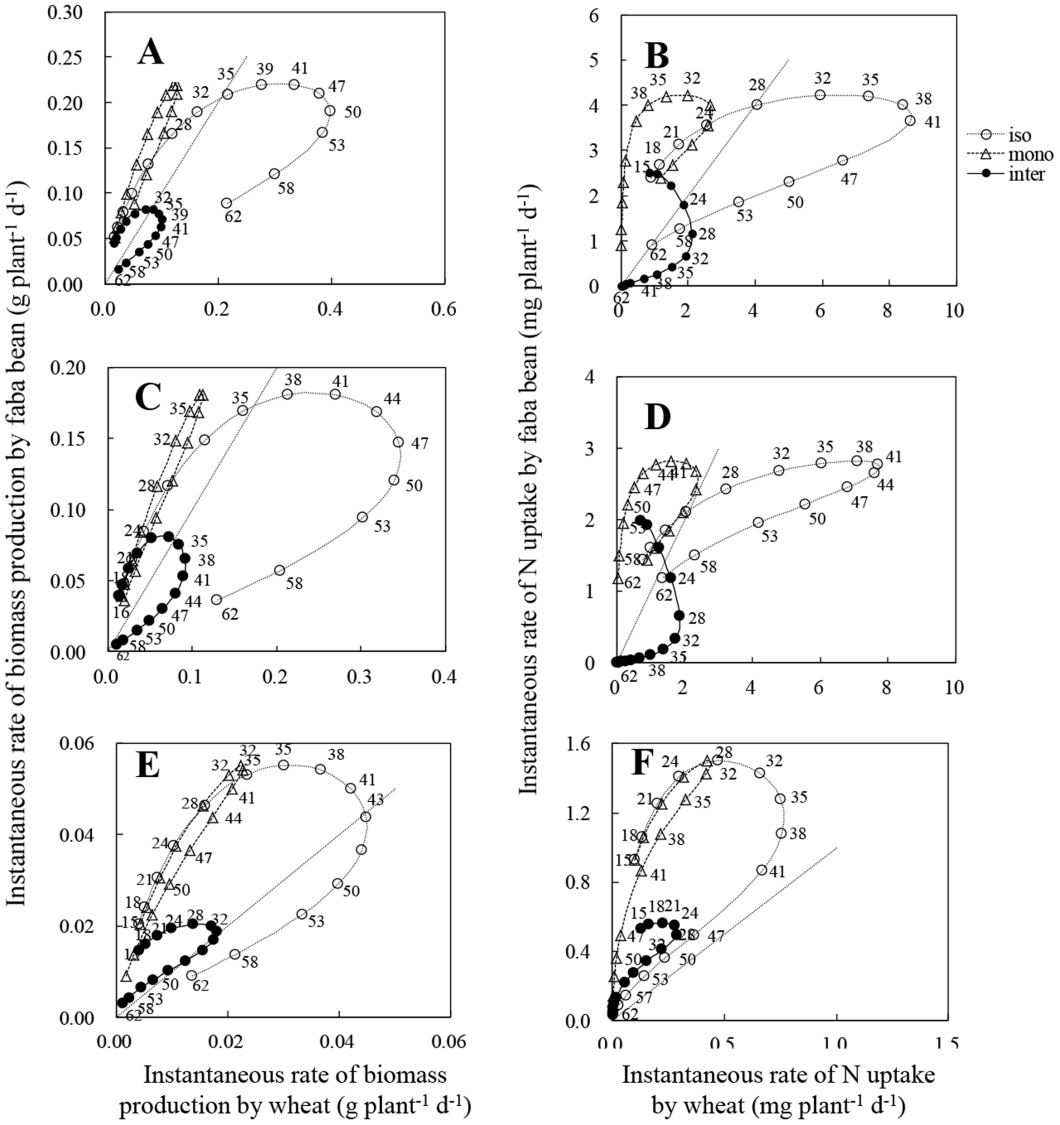
Trajectories of biomass production and nitrogen (N) uptake of total (A, B), shoot (C, D) and root (E, F) by isolated (iso: open circles and dashed line), monocropped (mono: open triangles and dashed curve), intercropped (inter: solid symbols and continuous line) wheat and according faba bean. Data was derived from the logistic equation using the mean of actual data at each sampling. Numbers near the curves represent the samplings time (day). The dashed line shows the 1∶1 relationships between faba bean and wheat.

The initial superiority of faba bean was evident in the trajectories of their instantaneous rates of biomass production and N uptake without competition (Fig. 6). At *c.* 35 DAS for total plant biomass, *c.* 36 DAS for shoot biomass and *c.* 43 DAS for root biomass, isolated wheat began to produce biomass faster than isolated faba bean did (Fig. 6A, C, E). With intraspecific competition, monocropped wheat lost superiority on instantaneous rate of biomass production compared with isolated faba bean. In the intercropping system, the initial superiority of faba bean was shorted by about 2–3 days for biomass production rate of total plant and shoots (Fig. 6A, C). In contrast, intercropped faba bean had a faster root production rate than intercropped wheat at most growth stages (Fig. 6E).

Isolated wheat had a superiority in N accumulation rate of total plant and shoot compared with isolated faba bean after *c.* 28 DAS and *c.* 24 DAS, which was earlier by 7 and 12 days than that of biomass production rate, respectively (Fig. 6B, D). Wheat roots always had a lower instantaneous rate of N accumulation than faba bean roots, regardless of cropping systems (Fig. 6F). Monocropped wheat had no superiority on N accumulation rate of total plant and shoot compared with isolated faba bean (Fig. 6B, D). With competition in intercropping system, wheat began to accumulate N faster, both in the total plant and in its shoot, than faba bean at *c.* 24 DAS and *c.* 23 DAS, respectively.

**Figure 7:**
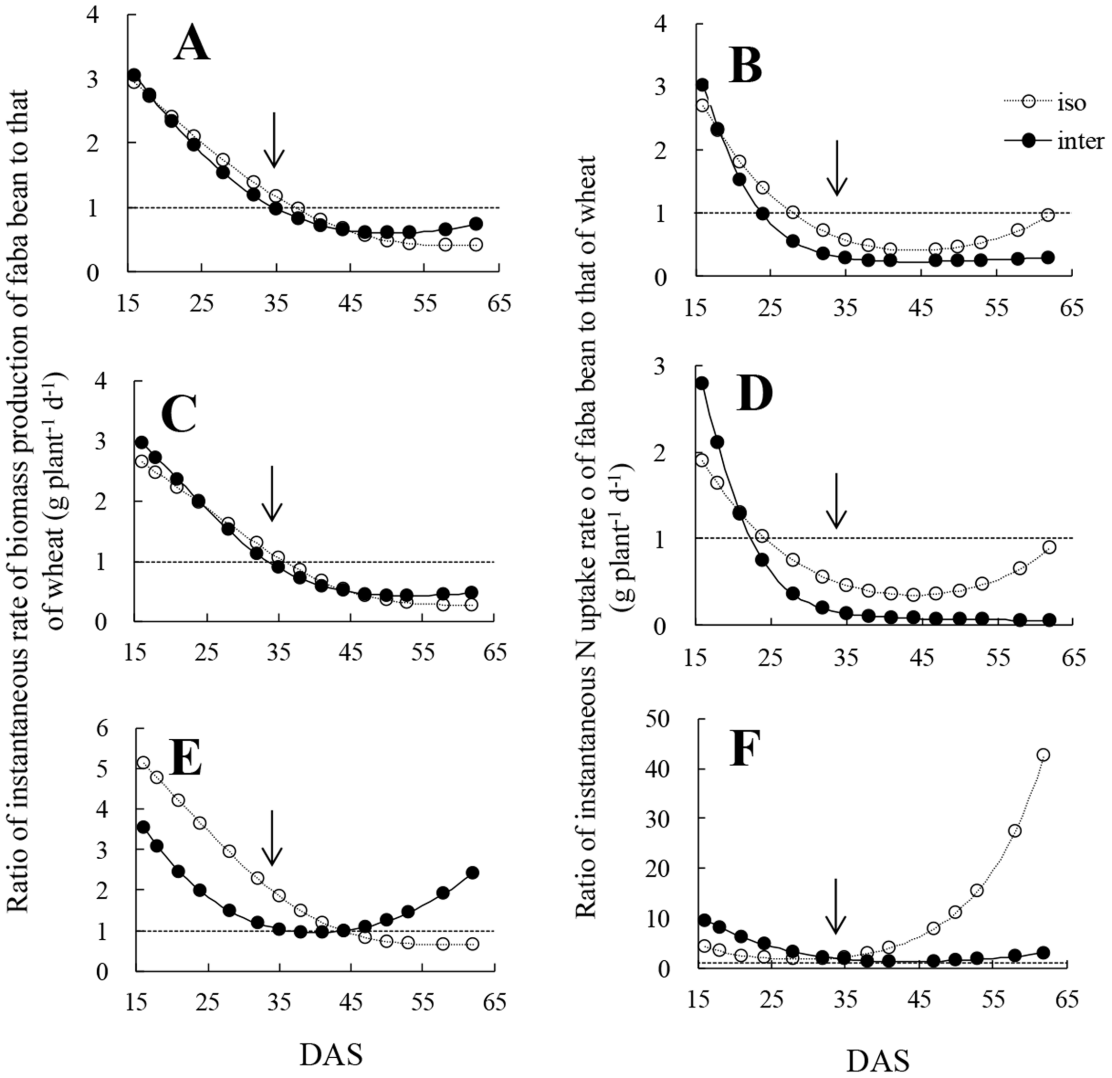
Ratio of instantaneous rate of biomass production of faba bean to that of wheat, and ratio of instantaneous nitrogen (N) uptake rate of faba bean to that of wheat in total (A, B), shoot (C, D) and root (E, F). Two lines in each graph indicate isolated (iso: open circle and dashed curves), intercropped (inter: solid symbols and continuous curves) wheat and according faba bean. The dashed lines indicate the y = 1. The arrows mean the date (33 DAS) of wheat stem elongation.

The ratio of the instantaneous rate of biomass production or N uptake of faba bean to that of wheat shows the relative intensity of growth and N uptake rate of two plant species. Competition stimulated the relative rate of biomass production of intercropped faba bean compared with that of isolated faba bean plants at *c.* 53 DAS for total plant and shoot, and at c. 47 DAS for roots (Fig. 7A, C, E). In contrast, intercropped wheat had not only an earlier but also a stronger intensive superiority in instantaneous rate of N uptake compared with intercropped faba bean (Fig. 7B, D, F).

## Discussion

### Plant growth and N uptake

The logistic model visualized competitive interaction in monocropping and intercropping. There was no competition for isolated plants, but intraspecific and interspecific competition arose with plant growth for monocropped and intercropped plants in the present study. The previous studies of intercropping usually focused on interspecific interactions, and ignored intraspecific interactions [2], [9]. In the present study, wheat was much more strongly and negatively affected by intraspecific competition than by interspecific competition by neighbouring faba bean. It was consistent with the previous study: with increasing plant density from 1.4 to 7 plants m^−2^, biomass of individual wheat decreased by more than 20% [19]. More than three times more N was absorbed by isolated than by monocropped wheat which caused a similar rate of biomass accumulation. In field experiments, Cereals often have a stronger competitive ability to take up N than legumes [20]. Nitrogen uptake by intercropped faba bean was suppressed by 63% compared with that of isolated plants. However, N uptake decreased only slightly in intercropped wheat compared with that of monocropped wheat. At *c.* 41 DAS, the total cumulative N uptake of wheat and faba bean per pot in intercropping system have reached up to 217 mg pot^−1^, which was approximately equal to the N supply capacity of 2 kg soil (original Nmin 36 mg plus N fertilizer addition 200 mg) in the present study. It indicated that the available N was almost exhausted by uptake of intercropped plants which can be confirmed by low Nmin around 3–7 mg kg^−1^ in soil and near-zero instantaneous N uptake rate of plants in intercropping system after *c.* 41 DAS. Thus, N demanded by plants growth in intercropping was mainly from re-utilization of plant N after *c.* 41 DAS in the present study.

The changing trends of instantaneous growth rate and N uptake appeared like a dome for both faba bean and wheat which is consistent with previous studies [15], [21]. Although other factors (light, temperature and nutrients other than N) were possibly also involved in competition in intercropping [22], competition for N was focused on as one of the major driving forces in this system. In pea/barley intercropping, the intensity of competition for soil N by plants determined grain yield and N accumulation. Nitrogen deficiency in the root medium reduces root elongation [23], and triggers the break-down of nucleic acids and proteins in leaves, causing leaf senescence and decreasing photosynthetic capacity [24]. Faster N uptake was associated with faster biomass production in the present study, and therefore isolated plants had a greater maximum instantaneous N uptake and biomass production rate than intercropped plants that suffered N deficiency caused by competition and depletion from neighbouring plants.

Based on fundamental characteristics of N uptake that different plant species reached maximum instantaneous N uptake at different times, isolated faba bean and wheat showed slightly temporal niche separation, which allowed them to acquire available resources at separated times [25]. Similar patterns of resource acquisition cause strong competition [22]. In the present study, some overlap of N uptake of faba bean and wheat occurred which caused competition between them. However, interactions reduced the extent of time overlap of N requirement in the intercropping system, and then alleviated the competition between different species. The mechanism of this effect is not clear. The detection of neighbouring plants [26], and auxin, as a plant hormone, plays an important role on regulation of plant growth [27], were expected to be involved in this process.

### Trajectories of biomass production and N uptake

The trajectories of biomass production and N uptake fully described the scale and nature of neighbour interaction, and showed that interaction strongly depended on sampling time. This trajectory analysis overcame the shortage of traditional experiments, with only one or two sampling times. Interactions between faba bean and wheat modified the dynamics of biomass production and N uptake compared with corresponding dynamics of isolated plants. As mentioned above, soil available N was close to be exhausted at *c.* 41DAS in intercropping system. Intra-competition strongly reduced instantaneous rate of biomass production and N uptake of individual wheat plants in the monocropping system which was possibly due to suppression of root growth before *c.* 41 DAS. Nitrogen accumulation of wheat had a positive correlation with root length [28]. Inter-specific competition had a greater effect on faba bean than on wheat, which was consistent with previous studies: strong competition from wheat roots tended to exhaust soil N and reduced N uptake of neighbouring faba bean from soil and enhanced the percentage of N derived from the atmosphere [8], [9]. Intercropped wheat increased its root to shoot ratio compared with that of intercropped faba bean (Table 1). Progressively larger investment in roots by wheat would have allowed it to gradually attain faster rates of N capture than that of faba bean which was similar to findings in previous studies [14], [29]. It was confirmed with a greater total N accumulation of intercropped wheat than that of intercropped faba bean by 32 mg pot^−1^ at final harvest in the present study.

Faba bean had superiority on biomass production and N uptake compared with wheat only at early stages in the intercropping system that resulted from fundamental characteristics of plant species which is consistent with the results of intercropped wheat and common vetch [30]. It is notable that the time at which isolated wheat overtook isolated faba bean in terms of cumulative or instantaneous N uptake preceded by 11–12 days the time at which it gained advantage in terms of biomass production. This indicates that the greater biomass production was due to faster N acquisition. Instantaneous N captured by isolated plants followed a similar, but wider and much rapider trajectory than that of the intercropped plants which indicates that the N uptake trajectory of the intercropped plants was not only determined by the nature of their interaction, but also by external conditions such as soil N availability. In the small pots used for plant growth, eventual exhaustion of available N was observed, for the intercropped treatment that had more plants than the isolated treatments. Earlier change-point in intercropping than in the isolated system was possibly due to strong competition of intercropped wheat for N. Before stem elongation, less light competition and early available N are strongly beneficial for crop growth [31]. During the period that wheat had a superiority compared with faba bean, the intensity of superiority was much higher on the instantaneous rate of N uptake in intercropping than in the isolated system. This indicates that inter-specific competition stimulates intercropped wheat to have greater intensive superiority compared with isolated wheat.

The information emerging from the present experiment shows that we can expect a temporal change of resource-capture dynamics if additional nutrients or other resources are considered at the same time. Moreover, it is necessary to extend this approach described above to explore competitive dynamics over a larger fraction of intercropped crops' long-term growth period in field. There is evidence that greater overyielding obtained through N fertilization or row ratio [32]. Therefore, it would be valuable to know how interactive dynamics between intercropped crops respond to local resources apply (spatial or temporal), planting density, neighbour size ect. Then the interspecific interactions would be managed by fertilizer application or rhizosphere management in order to maximize the facilitation at the same time as reducing competition.

## Conclusions

The logistic model visualized dynamics of biomass production and N uptake of faba bean and wheat with or without neighbours. When intercropped faba bean and wheat competed for N, there was a change-point of superiority on both biomass production and N uptake of faba bean and wheat with limited N supply. Faba bean had faster instantaneous rate of growth and N uptake only at early growth stages, and then was overtaken by wheat. Competition for soil N significantly modified these dynamics, not only temporally but also in intensity. This modeling methodology will be helpful to understand the underlying dynamic process of interaction in intercropping systems, and will be practicable to reveal the dynamic interaction of competitive resources utilization between intercropped plants in field.

## Supporting Information

S1 Table **P values using ANONA on biomass of plants.**
(DOCX)

S2 Table **P values using ANONA on N uptake of plants.**
(DOCX)
