# Correction: The Dynamic Process of Interspecific Interactions of Competitive Nitrogen Capture between Intercropped Wheat (*Triticum aestivum* L.) and Faba Bean (*Vicia faba* L.)

**DOI:** 10.1371/journal.pone.0119659

**Published:** 2015-03-24

**Authors:** The PLOS ONE Staff

All authors are incorrectly listed as contributing equally to the published paper.
