# Supplementary material for: The Dynamic Process of Interspecific Interactions of Competitive Nitrogen Capture between Intercropped Wheat (Triticum aestivum L.) and Faba Bean (Vicia faba L.)
Source: PLoS One. 2014 Dec 26;9(12):e115804. doi: 10.1371/journal.pone.0115804 (PMC4277399; doi:10.1371/journal.pone.0115804)
Supplement: S1 Table — P values using ANONA on biomass of plants. (DOCX) [file pone.0115804.s001.docx]

**S1 Table.** ***P* values using ANONA on biomass of plants.**

| Species |  | 15 | 18 | 21 | 24 | 28 | 32 | 35 | 38 | 41 | 47 | 50 | 53 | 58 | | 62 |
| --- | --- | --- | --- | --- | --- | --- | --- | --- | --- | --- | --- | --- | --- | --- | --- | --- |
|  | total | 0.3692 | 0.2748 | 0.5424 | 0.1156 | 0.3216 | 0.0007 | 0.0182 | 0.0430 | 0.0003 | <0.0001 | <0.0001 | <0.0001 | <0.0001 | <0.0001 | |
| Wheat | shoot | 0.2608 | 0.2183 | 0.0810 | 0.6725 | 0.1490 | 0.0006 | 0.0033 | 0.0370 | <0.0001 | <0.0001 | <0.0001 | <0.0001 | <0.0001 | <0.0001 | |
|  | root | 1.0000 | 0.1944 | 0.5502 | 0.1014 | 0.8374 | 0.0084 | 0.2864 | 0.0691 | 0.5530 | 0.0024 | 0.0016 | 0.0024 | 0.0086 | 0.0114 | |
|  | total | 0.5122 | 0.8556 | 0.0687 | 0.2676 | 0.3814 | 0.0020 | 0.0005 | 0.0217 | 0.0003 | <0.0001 | 0.0003 | 0.0166 | 0.0063 | 0.0004 | |
| Faba bean | shoot | 0.3782 | 0.7944 | 0.0455 | 0.4306 | 0.3078 | 0.0032 | 0.0184 | 0.0406 | 0.0331 | <0.0001 | 0.0002 | 0.0102 | 0.0063 | 0.0032 | |
|  | root | 0.8593 | 1.0000 | 0.2679 | 0.1120 | 0.0604 | 0.0165 | 0.0078 | 0.2038 | 0.0112 | 0.0004 | 0.0026 | 0.0576 | 0.0122 | 0.0036 | |

Note: Each value means the *P* values compared the biomass between the isolated wheat or faba bean and monocropped or intercropped wheat or faba bean in total, shoot and root.
