# Supplementary material for: The Dynamic Process of Interspecific Interactions of Competitive Nitrogen Capture between Intercropped Wheat (Triticum aestivum L.) and Faba Bean (Vicia faba L.)
Source: PLoS One. 2014 Dec 26;9(12):e115804. doi: 10.1371/journal.pone.0115804 (PMC4277399; doi:10.1371/journal.pone.0115804)
Supplement: S2 Table — P values using ANONA on N uptake of plants. (DOCX) [file pone.0115804.s002.docx]

**S2 Table. *P* values using ANONA on N uptake of plants.**

| Species |  | 15 | 18 | 21 | 24 | 28 | 32 | 35 | 38 | 41 | 47 | 50 | 53 | 58 | 62 |
| --- | --- | --- | --- | --- | --- | --- | --- | --- | --- | --- | --- | --- | --- | --- | --- |
|  | total | 0.4626 | 0.0202 | 0.0139 | 0.9508 | 0.1148 | 0.0177 | 0.0222 | 0.0052 | <0.0001 | 0.0003 | <0.0001 | 0.0004 | 0.0002 | <0.0001 |
| Wheat | shoot | 0.2620 | 0.3446 | 0.0213 | 0.0344 | 0.8620 | 0.1705 | 0.0285 | 0.0031 | <0.0001 | 0.0003 | <0.0001 | 0.0003 | 0.0002 | <0.0001 |
|  | root | 0.6818 | 0.0537 | 0.6376 | 0.9019 | 0.0865 | 0.0769 | 0.3843 | 0.0895 | 0.0186 | 0.0021 | 0.0002 | 0.0010 | <0.0001 | 0.0016 |
|  | total | 0.9286 | 0.5684 | 0.0070 | 0.4399 | 0.0097 | 0.0019 | 0.0131 | 0.0395 | 0.0369 | 0.0018 | 0.0033 | 0.0878 | 0.0173 | 0.0013 |
| Faba bean | shoot | 0.8015 | 0.2075 | 0.0129 | 0.0016 | 0.6208 | 0.0010 | 0.0923 | 0.1071 | 0.1080 | 0.0020 | 0.0024 | 0.1011 | 0.0134 | 0.0024 |
|  | root | 0.4685 | 0.0222 | 0.0283 | 0.1505 | 0.0378 | 0.0078 | 0.0079 | 0.1381 | 0.0381 | 0.0067 | 0.0057 | 0.0834 | 0.0298 | 0.0080 |

Note: Each value means the *P* values compared the N uptake between the isolated wheat or faba bean and monocropped or intercropped wheat or faba bean in total, shoot and root.
